# The reduction of LEDD leads to visual dysfunction in patients with PD after STN-DBS: a randomized clinical trial

**DOI:** 10.1097/JS9.0000000000002018

**Published:** 2024-08-05

**Authors:** Jinxing Sun, Shengmei Ma, Zhenke Li, Junheng Jia, Qianqian Wu, Ying Hou, Hong Wang, Qi Wang, Guangjian Zhang, Zhimin Zhao, Bin Huang, Xiangyu Ma, Xingang Li, Weiguo Li, Chao Zhang

**Affiliations:** a Department of Neurosurgery, Qilu Hospital of Shandong University; b Institute of Brain and Brain-Inspired Science, Shandong University; c Department of Radiology, Qilu Hospital of Shandong University; d Department of Neurology, Qilu Hospital of Shandong University; e Department of Ophthalmology, Qilu Hospital of Shandong University; f Department of Gerontology, Shandong Provincial Qianfoshan Hospital, Jinan; g Department of Neurology, Weifang People’s Hospital, Weifang, China; h National Medicine-Engineering Interdisciplinary Industry-Education Integration Innovation Platform, China

**Keywords:** antiparkinsonian medications, Parkinson’s disease, RNFL thickness, STN-DBS, visual dysfunction

## Abstract

**Background:**

Medication adjustment after deep brain stimulation (DBS) of the subthalamic nucleus (STN) in patients with Parkinson’s disease (PD) may influence visual function. However, no clinical trials have been designed specifically to investigate this effect.

**Objectives:**

To compare the effects of levodopa-equivalent daily dose (LEDD) reduction and non-reduction on visual function in patients with Parkinson’s disease (PD) following STN-DBS.

**Methods:**

This was a multi-center, prospective, randomized, double-blinded clinical trial. A total of 208 patients with Parkinson’s disease were referred for DBS between June 2019 and July 2021 and analyzed between June 2023 and July 2023. STN-DBS was performed, and the LEDD was reduced in one study arm but not in the other. The primary outcome measure was visual impairment in Parkinson’s disease questionnaire (VIPD-Q) with or without LEDD reduction 12 months postoperatively, and the secondary outcomes included retinal nerve fiber layer (RNFL) thickness, vessel density, eye-tracking system results, contrast sensitivity and visual field.

**Results:**

During the short-term follow-up, DBS implantation and stimulation did not significantly affect visual function (VIPD-Q, baseline vs. 1 month, 9.269±8.385 vs. 8.938±7.666, Mann–Whitney U tests; *P*=0.6746). In the long-term follow-up, the reduction group demonstrated a significant decline in visual function, RNFL thickness, and vessel density after STN-DBS compared with the control group without STN-DBS (*P*<0.001).

**Conclusions:**

Visual dysfunction, particularly a thinner RNFL and lower vessel density, is related to LEDD reduction after STN-DBS. Prolonged administration of dopamine-mimetic drugs prevents visual symptoms. Thus, physicians should consider LEDD adjustment when patients report visual dysfunction before surgery or severe visual symptoms after STN-DBS.

## Introduction

Highlights

- In this clinical trial, ophthalmological symptoms worsened in 44.8% of the patients with PD after STN-DBS (vs. 4% of the controls without STN-DBS).
- LEDD reduction was correlated with visual dysfunction.
- The visual saccades were influenced by DBS rather than by levodopa.
- Our findings focus on the visual symptoms in patients with PD and provide novel insights into the potential influence of levodopa reduction after STN-DBS.

Parkinson’s disease (PD) has a large effect on society; it affected ~6.1 million people worldwide in 2016^1^. However, the incidence and prevalence of PD have increased rapidly in the past two decades^2^. Motor outcomes have been extensively studied. However, non-motor symptoms (NMS) in PD have not received sufficient attention. NMS encompasses various clinical manifestations including cognitive dysfunction, behavioral changes, hyposmia, dysautonomia, and visual dysfunction^3,4^.

Vision is an important determinant of quality of life because the screen time of individuals has dramatically increased. Persons with PD are at an increased risk for visual symptoms compared with the general population^5^. However, ophthalmologic symptoms are underreported by patients with PD and often overlooked by their treating physicians.

The number of patients undergoing deep brain stimulation (DBS) has increased exponentially worldwide over the past few years^6,7^. DBS is commonly indicated for the treatment of movement disorders such as PD, tremor, and dystonia^8^. However, its effect on ophthalmologic symptoms has rarely been reported. Even though circuits involved in the control of eye movements traverse the basal ganglia and are, therefore, likely to be influenced by DBS, the integration of DBS with eye movement analysis in research has been sporadic. Recent studies demonstrate that DBS in the subthalamic nucleus (STN) can enhance smooth pursuit in Parkinson’s disease. Furthermore, STN-DBS has been shown to modulate visuospatial attention effectively^9^. Simultaneous bilateral STN and PPN DBS produced greater improvements in mean latencies, velocities, and accuracies for visually guided saccades and antisaccades compared to the DBS-off condition^10^. Although DBS can improve saccadic and oriented gaze movements^11–13^, a few patients report severe visual dysfunction after STN-DBS^14^. Therefore, a study with a larger sample size is required to determine whether visual dysfunction after STN-DBS is a common or chance event and further analyze the reasons behind it.

After STN-DBS, patients with PD could reduce their levodopa (LD)-equivalent dose (LEDD) by an average 50% based on the consideration of lowering dyskinesias risk^15^. However, LEDD improves the central visual acuity by an average of six lines on the Snellen acuity chart and can promote neuroprotection of the maculopapular retinal ganglion cell fibers^16,17^. This necessitates determining whether the visual deterioration can be attributed to the reduction of medication. No clinical trials have evaluated the effects of antiparkinsonian medications on visual function after STN-DBS.

This study aimed to compare the effects of LEDD reduction and non-reduction on visual function in patients with PD after STN-DBS. We hypothesized that, as opposed to LEDD reduction, a stable dose of medication may be more beneficial in avoiding visual impairment after STN-DBS.

## Methods

### Participants

Patients were eligible for enrollment if they had received a clinical diagnosis of idiopathic PD according to the British Parkinson’s Disease Society Brain Bank criteria. The specific inclusion criteria were: (1) age older than 18 years; (2) idiopathic PD with at least two of three signs (resting tremor, rigidity, and bradykinesia); (3) at least one of four symptoms (severe response fluctuations, dyskinesias, painful dystonia, or bradykinesia), despite optimal pharmacological treatment; (4) a life expectancy of at least 2 years; and (5) agreed to accept DBS surgery.

Patients were ineligible if they had any of the following exclusion criteria: (1) legally incompetent; (2) secondary parkinsonism; (3) previous neurosurgery for PD (e.g. DBS, pallidotomy, thalamotomy); (4) contraindications for DBS surgery, such as a physical disorder that made surgery hazardous; (5) Hoehn and Yahr stage 5 at the best moment during the day; (6) severe cognitive impairment indicated by a Mattis Dementia Rating Scale score less than 120; (7) current depression or psychosis; (8) lack of written informed consent; (9) glaucoma, intraocular surgery, diabetes or other diseases affecting the visual field or neurologic systems; or (10) current use of medications affecting visual function.

### Trial design and randomization

This multi-center, double-blinded, randomized control study is part of a larger study on non-motor symptoms after DBS in patients with PD. This work has been reported in line with Consolidated Standards of Reporting Trials (CONSORT, Supplemental Digital Content 1, http://links.lww.com/JS9/D246, Supplemental Digital Content 2, http://links.lww.com/JS9/D247) Guidelines^18^.

Patients who were candidates for STN-DBS underwent DBS according to a previous study^19^. Quadripolar electrodes (model 3389) were connected to an implantable pulse generator (Activa PC, Medtronic, Dublin, Ireland) and implanted in the candidates. Randomization, monitoring, and data management were performed locally. Randomization was performed using an online list randomizer (https://www.random.org/lists).

**Figure 1:**
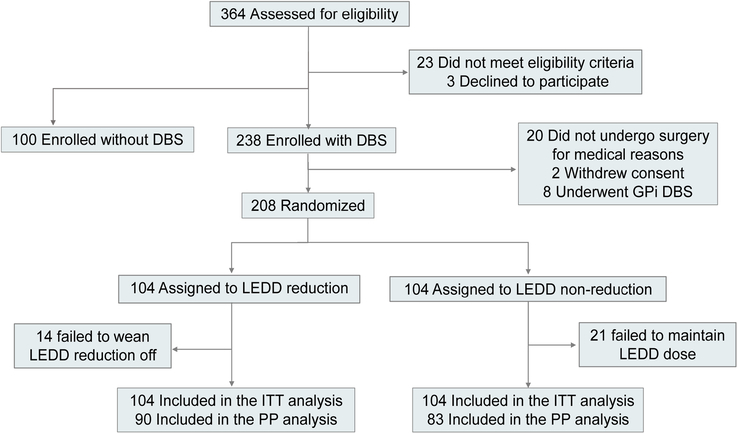
Study flowchart. DBS, deep brain stimulation; LEDD, levodopa-equivalent dose; ITT, Intention-to-treat; PP, Per-Protocal.

Written informed consent was obtained from all participants. After the baseline measurements, we informed the eligible patients of the assigned randomization using sealed opaque envelopes marked with inpatient numbers (Fig. 1). The participants were randomized to receive either an unchanged LEDD after surgery (the non-reduction group), or a reduced LEDD after surgery (the reduction group).

### Intervention

Patients with STN-DBS and physicians responsible for evaluating visual function were blinded to randomization. A professional neurologist was unblinded and in charge of reducing medication during the visit of enrolled patients. Patients in the non-reduction group were expected to remain on their current LEDD for 12 months, while patients in the reduction group gradually reduces the LEDD according to the expert consensus (replaced by placebo, which was provided by a qualified manufacturer and had a similar shape and weight with common antiparkinsonian medications). For LEDD reduction processes, the 1–3 months after the implantation of DBS is the key period and the process of mutual adaptation between the patient and stimulation. Due to the short half-life of levodopa, we would first reduce the dose of levodopa to find the most appropriate stimulation parameters and assess stimulation efficacy. Subsequently, we would taper or discontinue dopamine receptor (DR) agonists and replace them with levodopa. As the stimulation parameters increase and the symptoms further improve, the dose of the levodopa would be further reduced until stable symptom control was achieved. After the optimal parameter settings are stabilized, we would replace levodopa by a long-acting DR agonists according to the levodopa equivalent. Patients volunteered to have extra visits 24 months after the endpoint. The dose intensity of LEDD was calculated based on the equation in the publication of Jost *et al.* ^20^. Patients volunteered to have extra visits 24 months after the endpoint.

### Outcomes

The primary outcome was the VIPD-Q score 12 months postoperatively, and the secondary outcomes included retinal nerve fiber layer (RNFL) thickness, vessel density, eye-tracking system results, contrast sensitivity, and visual field deviation.

We used the visual impairment in Parkinson’s disease questionnaire (VIPD-Q)^21^ to assess ophthalmologic symptoms (SDC, Supplementary Figure 1A, Supplemental Digital Content 3, http://links.lww.com/JS9/D248), Unified Parkinson’s Disease Rating Scale (UPDRS III), Montreal Cognitive Assessment (MoCA), Mini-Mental State Examination (MMSE), Hospital Anxiety and Depression Scale (HAD) Anxiety subscale, and HAD-depression subscale scores in patients with PD at 2 days pre-DBS implantation and 1, 6, and 12 months post-DBS implantation. The questionnaire was administered at three university hospitals and scored by three clinicians (one ophthalmologist and two neurologists) between June 2019 and July 2021.

The participants underwent neuro-ophthalmological examinations, including ocular fundus photography, eye-tracking, and optical coherence tomography (OCT) in both eyes at 2 days pre-DBS implantation and 1, 6, and 12 months post-DBS implantation, and the mean values were calculated for comparison.

### Ethical considerations

The protocol was approved by the local ethics committee and was conducted in accordance with the principles of the Declaration of Helsinki.

### Optical coherence tomography and ocular fundus photography

The RNFL and vessel density were measured using a Spectralis OCT device (CIRRUS 5000; Carl Zeiss, Oberkochen, Germany; Supplementary Figure 1B, Supplemental Digital Content 3, http://links.lww.com/JS9/D248). The scans were performed by an identical operator. The images were acquired using TruTrack eye-tracking technology, which recognizes, locks, and follows the patient’s retina. We used Spectralis segmentation software to determine the mean retinal thickness. At least one reliable image of the ocular fundus per eye was acquired for each patient (Supplementary Figure 1C, Supplemental Digital Content 3, http://links.lww.com/JS9/D248).

### Visual-guided and memory-guided saccade tasks

Participants completed two oculomotor tasks: a visual-guided saccade (VGS) and a memory-guided saccade (MGS). The two tasks differed in the timing of the target offset (Supplementary Figure 1D, Supplemental Digital Content 3, http://links.lww.com/JS9/D248). Each task began with the participant fixating on a central point (0° visual angle) for a variable 2–3 s to limit anticipatory movements, followed by a peripheral target turned on at 5°, 10°, 20°, or 30° to the left or right. During the VGS task, participants had to make a saccade quickly to a new position. During the MGS task, the target disappeared after 0.05 s, and the subject had to quickly make a saccade to the remembered location of the target after 2–3 s. If the velocity and acceleration exceeded the threshold (28°/s and 90°/s2, respectively), we assumed that a saccade occurred. We analyzed the saccades based on previous descriptions^12^.

### Visual function measures

#### Contrast sensitivity

We used the functional acuity contrast test (F.A.C.T.; Stereo Optical, Chicago, IL) to obtain the contrast sensitivity score at five spatial frequencies as follows: 1.5, 3, 6, 12, and 18 cycles/degree^22^. The Functional Vision Analyzer presents a sinusoidal grating in the form of a Gabor patch. Each spatial frequency is presented as a series of nine patches with 0.15 log units, or 50% contrast loss between consecutive patches. The participant was required to point out the direction of the gratings that tilted to the left (+15°), right (−15°), or upright (0°). The contrast of the last grating that the observer can distinguish is the contrast sensitivity score.

#### Visual field

We used a Humphrey visual field analyzer (Zeiss Meditec) to assess the automated visual field sensitivity in each eye^22^. First, participants need to ensure that their eyes are in the same position by staring at a small light located in the center. Then, tests are conducted in different directions of the field of view until the threshold appears. The results obtained included (1) mean deviation and (2) pattern standard deviation reported in decibels (dB). Mean deviation (MD) refers to the mean deviation of the participants’ results compared to the expected results of the age-matched healthy person database. As the entire field deteriorates, these values become increasingly negative. The pattern standard deviation (PSD) describes poor focusing. This is determined by comparing the differences between adjacent points. The higher the value, the greater the number of focus defects.

### DBS leads localization and VTA estimation

DBS leads were localized and reconstructed with the Lead-DBS toolbox (www.lead-dbs.org). The detailed processing pipeline has been described elsewhere^23^. The estimation of tissue volume (VTA) activation was carried out utilizing the finite element method, as introduced by Horn and colleagues, based on clinically optimized stimulation parameters and analyzed in accordance with a prior study^24^. Electrodes reconstructed in standard stereotactic space projected on the STN as implemented in the DISTAL Atlas (Ewert 2017)^25^.

### Statistical analysis

Based on earlier treatment results^26^, we calculated that a sample size of 126 patients (63 each in both groups) would provide 80% power to reject the null hypothesis of equal means for a mean difference of −5 (10–15) in the primary outcome (VIPD-Q scores between reduction vs. non-reduction), with standard deviation of 10 for both the reduction and non-reduction groups at a two-sided alpha of 0.05. Considering the anticipated dropout rate of 20%, the total sample size required was 158 (79 each in both group).

Patients with LEDD reduction were compared with non-reduction controls using χ^2^ tests and Mann–Whitney U tests for categorical variables (sex and visual aid use) and non-parametric continuous variables [age, disease duration, VIPD-Q, Non-Motor Symptoms Scale, Excessive Daytime Sleepiness Questionnaire, Montreal Cognitive Assessment, MMSE, Hospital Anxiety and Depression Scale-anxiety subscale, Hospital Anxiety and Depression Scale-depression subscale, Unified Parkinson’s Disease Rating Scale Part III (UPDRS III), and LEDD], respectively. Similar statistical methods were also applied to compare patients with STN-DBS and those without STN-DBS. The two-tailed paired Student’s *t*-test was used to compare the continuous variables in accordance with the normal distribution: the results of the RNFL, optical coherence tomography angiography (OCT-A), visual field, contrast sensitivity, saccade tasks, stimulation parameters and VTA. The normal distribution of the *t*-test was examined by the Kolmogorov–Smirnov test, and the *P* value was over 0.10.

Linear models and Spearman’s ρ values were used to explore the correlations between the LEDD, VIPD-Q, and RNFL. Finally, we performed multiple univariate logistic regression analysis to identify the risk factors for visual dysfunction (independent variables explored: age, disease duration, UPDRS III, LEDD, and gender randomization). The false discovery rate (FDR) was used to correct the *P* value for multiple comparisons. Analysis of covariance (ANCOVA) was used to evaluate the homogeneity of the primary and secondary outcome across multiple centers, and the *P* value was over 0.10. No data were missing in this study. Statistical analysis was primarily performed by intention-to-treat and secondarily by protocol. Prism software (version 9.3.0; San Diego, CA, USA) was used for statistical analyses.

## Results

### Participant characteristics

A total of 338 participants were enrolled between June 2019 and July 2021; 208 patients who underwent STN-DBS were randomized to receive medication reduction (*n*=104) or no reduction (*n*=104) (Fig. 1). In addition, 100 age-matched patients who did not undergo DBS were enrolled in the control group without STN-DBS to exclude visual impairments caused by STN-DBS. The number of patients included in each center was showed in Supplementary Table 1, Supplemental Digital Content 3, http://links.lww.com/JS9/D248. Fourteen participants in the reduction group did not have a reduction in the LEDD due to intolerance of PD symptom deterioration associated with levodopa tapering, and 21 patients in the non-reduction group did not maintain their medication dose due to medication side effects (e.g. levodopa-induced dyskinesia); therefore, we performed an intention-to-treat analysis and a per-protocol analysis.

None of the baseline characteristics differed significantly between the groups before surgery (Table 1 and Supplementary Table 2, Supplemental Digital Content 3, http://links.lww.com/JS9/D248). The efficacy of STN-DBS across the entire cohort was underscored by a notable mean decrease in the UPDRS-III total score (from baseline to 12 months in PD patients with DBS, 20.18±10.47–12.73±5.61, using Mann–Whitney U tests, *P*<0.01). There was no heterogeneity among the three centers for all of the primary and secondary outcomes (ANCOVA, *P*>0.10).

**Table 1:** Baseline characteristics of the intention-to-treat and per-protocol cohort before surgery.

	Intention-to-treat population			Per-protocol analysis		
	PD (reduction) (*n*=104)	PD (non-reduction) (*n*=104)	*P*	PD (LEDD reduction) (*n*=90)	PD (LEDD non-reduction) (*n*=83)	*P*
Men, *n* (%)	50 (48.08)	60 (57.69)	0.1648	43 (47.78)	40 (48.19)	0.9565
Age, (year)	62.82±8.97	61.75±9.24	0.2888	62.21±9.15	61.16±9.18	0.2734
Disease duration, (year)	9.08±4.39	9.25±4.95	0.9917	8.87±4.09	9.3±5.29	0.9374
NMSS	56.78±42.30	54.87±43.38	0.6832	55.26±38.44	57.51±41.17	0.7105
UPDRS-III (med on)	22.33±11.39	21.05±11.12	0.4405	21.64±11.64	23.12±12.00	0.4678
MoCA	25.53±1.36	25.61±1.46	0.5718	25.66±1.36	25.6±1.29	0.6699
MMSE	27.99±1.62	27.84 ±1.61	0.6733	28.08±1.68	27.83±1.66	0.4964
HAD-A	5.43±3.27	5.82±3.09	0.2911	5.31±3.27	5.86±3.00	0.1890
HAD-D	5.11±2.35	4.92±2.57	0.3888	5.06±2.30	4.60±2.84	0.1495
Uses visual aid, *n* (%)	30 (28.8)	32 (30.8)	0.7618	27 (30.0)	26 (31.3)	0.8502
LEDD (mg)	791.1±294.2	735.3±266.2	0.1553	744±258.7	793.5±287.2	0.2387
VIPD-Q	9.32±8.44	9.22±8.37	0.7771	9.56±8.78	9.48±8.56	0.9518
RNFL	83.27±4.57	82.85±5.00	0.5279	84.34±5.59	85.73±5.27	0.0949
OCT-A	8.27±0.58	8.36±0.64	0.2892	8.43±0.48	8.59±0.61	0.0559
Visual field mean deviation, dB/y	−0.08 (1.08)	−0.115 (2.08)	0.8791	−0.07 (1.21)	−0.131 (2.14)	0.8160
Visual field pattern standard deviation, dB/y	−0.06 (0.54)	−0.14 (0.62)	0.3222	−0.05 (0.38)	−0.16 (0.67)	0.1816
Contrast sensitivity (2 units/y=0.3 log unit change)						
1.5 cpd	−1.45 (7.67)	−1.20 (7.12)	0.8078	−1.57 (7.46)	−1.32 (7.84)	0.8301
3 cpd	−0.44 (9.45)	−0.31 (14.16)	0.9380	−0.41 (8.96)	−0.37 (12.87)	0.9810
6 cpd	−0.73 (14.1)	−1.08 (16.48)	0.8694	−0.69 (13.5)	−1.11 (15.34)	0.8484
12 cpd	−1.52 (8.52)	−1.33 (8.64)	0.8733	−1.48 (8.61)	−1.41 (8.54)	0.9573
18 cpd	−0.36 (4.03)	0.44 (3.52)	0.1289	−0.39 (4.54)	0.48 (3.73)	0.1723
Visual guide saccade						
Saccade latency	297.7±65.43	288.1±67.71	0.2997	301.5±64.62	292.8±66.58	0.3845
Saccade error rate	17.84±13.27	18.05±13.84	0.9112	16.87±14.22	17.65±14.32	0.7199
Saccade reaction time	228.8±39.74	232.2±41.14	0.5451	232.4±38.56	228.4±47.25	0.5413
Memory guide saccade						
Saccade latency	401.7±34.10	399.1±38.44	0.6064	407.7±33.55	399.2±36.74	0.1136
Saccade error rate	19.25±10.84	18.40±11.80	0.5891	20.27±10.71	17.49±12.75	0.1213
Saccade reaction time	353.1±78.76	343.1±81.21	0.3684	356.8±74.55	348.1±82.27	0.4666

cpd, cycles per degree; HAD, Hospital Anxiety and Depression Scale; LEDD, levodopa-equivalent dose; MoCA, Montreal Cognitive Assessment; MMSE, Mini-Mental State Examination; NMSS, Non-Motor Symptoms Scale; OCT-A, optical coherence tomography angiography; PD, Parkinson’s disease; RNFL, Retinal Nerve Fiber Layer; UPDRS III, Unified Parkinson’s Disease Rating Scale Section III; VIPD-Q, Visual Impairment in Parkinson’s Disease Questionnaire.

Intention-to-treat and per-protocol analyses suggested that the stimulation parameters did not differ between the treatment groups (*t*-test, *P*>0.05, Supplementary Table 3, Supplemental Digital Content 3, http://links.lww.com/JS9/D248).

Intent-to-treat and per-protocol analyses showed significant reductions in LEDD at endpoint in the reduction group compared to the non-reduction group (Mann–Whitney U tests, *P*<0.001, Table 2). Table 2 also summarizes the primary and secondary outcomes, including the VIPD-Q score, saccades in visual tasks, OCT-A examination results, mean visual field deviation, pattern standard deviation, and contrast sensitivity at five spatial frequencies.

**Table 2:** LEDD, primary and secondary outcomes at 12 months after surgery.

	Intention-to-treat population			Per-protocol analysis		
	PD (reduction) (*n*=104)	PD (non-reduction) (*n*=104)	*P/Q* value	PD (reduction) (*n*=90)	PD (non-reduction) (*n*=83)	*P/Q* value
LEDD (mg)	458.8±177.6	755.2±270.3	<0.001	444.5±182.7	788.9±272.4	<0.001
VIPD-Q	14.07±8.99	10.05±8.37	<0.001	15.13±9.14	10.84±8.86	0.002
RNFL thickness	76.84±8.02	82.43±5.03	<0.001	74.73±8.55	85.82±5.74	<0.001
Vessel density	8.33±0.68	7.49±0.66	<0.001	8.72±0.77	7.58±0.72	<0.001
Visual field						
Visual field mean deviation, dB/y	−2.14 (1.21)	−0.106 (0.23)	<0.001	−2.31 (1.64)	−0.187 (0.31)	<0.001
Visual field pattern standard deviation, dB/y	1.09 (0.46)	−0.10 (0.56)	<0.001	1.15 (0.52)	−0.12 (0.49)	<0.001
Contrast sensitivity (2 units/y=0.3 log unit change)						
1.5 cpd	−2.16 (9.45)	−0.88 (9.04)	0.3194	−2.48 (9.27)	−0.76 (9.53)	0.2307
3 cpd	−2.61 (6.56)	−0.51 (6.08)	0.2917	−2.59 (6.42)	−0.57 (6.14)	0.0603
6 cpd	−2.03 (6.58)	0.23 (6.08)	0.0288	−2.33 (6.23)	0.32 (6.05)	0.0255
12 cpd	−2.78 (5.82)	−0.78 (5.48)	0.0288	−2.76 (5.57)	−0.74 (5.35)	0.0405
18 cpd	−0.62 (6.35)	0.23 (4.32)	0.3194	−0.69 (6.74)	0.43 (4.58)	0.2307
Visual guide saccade						
Saccade latency	301.2±66.35	292.5±67.45	0.8523	312.3±67.55	295.5±66.73	0.3063
Saccade error rate	15.92±12.82	16.26±13.47	0.8523	16.32±13.41	15.74±14.46	0.7846
Saccade reaction time	229.6±41.08	232.8±40.29	0.8523	228.3±42.32	234.7±41.59	0.4767
Memory guide saccade						
Saccade latency	399.8±31.75	394.3±48.08	0.4973	396.4±34.83	391.5±47.68	0.4388
Saccade error rate	20.35±10.97	19.71±11.69	0.6843	21.46±10.83	18.72±12.54	0.3753
Saccade reaction time	354.3±81.20	338.9±81.38	0.4973	357.4±82.27	343.7±82.35	0.4134

cpd, cycles per degree; LEDD, levodopa-equivalent dose; PD, Parkinson’s disease; RNFL, retinal nerve fiber layer; VIPD-Q, Visual Impairment in Parkinson’s Disease Questionnaire.

### VIPD-Q score and LEDD reduction

**Figure 2:**
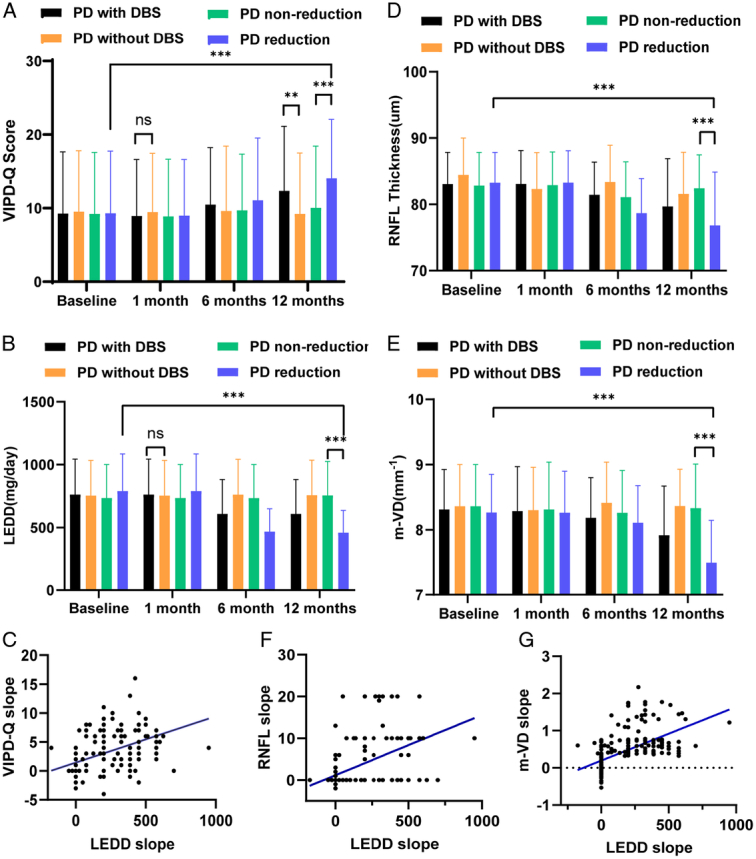
The figures show the relationship between LEDD reduction and VIPD-Q, RNFL, VD. Graph shows mean slope of averaged VIPD-Q (A) and LEDD (B) at baseline, 1, 6, and 12 months in different groups. (C) The plot shows that change over time in LEDD (LEDD slope) was positively associated with change over time in VIPD-Q (VIPD-Q slope). Graph shows mean slope of averaged RNFL (D) and microvascular density (E) in the fundus of patients with PD at baseline, 1, 6, and 12 months in different groups. The plot shows that change over time in LEDD (LEDD slope) was positively associated with change over time in RNFL thickness (RNFL thickness slope) (F) and microvascular density in the fundus (m-VD slope) (G). DBS, deep brain stimulation; LEDD, levodopa-equivalent dose; PD, Parkinson’s disease; RNFL, retinal nerve fiber layer; VIPD-Q, Visual Impairment in Parkinson’s Disease Questionnaire.

DBS implantation did not cause visual dysfunction in patients with PD one month after surgery (PD with DBS vs. PD without DBS, VIPD-Q: Mann–Whitney U tests, *P*=0.9216, Fig. 2A) when the medication was not adjusted (PD with DBS vs. PD without DBS, LEDD: Mann–Whitney U tests, *P*=0.7991, Fig. 2B). Twelve months later, the ophthalmological symptoms worsened (>50% VIPD-Q score increase) in 44.8% of the patients with STN-DBS (vs. 4% of patients without STN-DBS; χ^2^ test: *P* <0.001). Additionally, VIPD-Q scores increased dramatically in the DBS group compared to those in the control group without STN-DBS (PD with DBS vs. PD without DBS, VIPD-Q: Mann–Whitney U tests, *P*<0.001, Fig. 2A). Patients with PD in the medication reduction group demonstrated reduced LEDD from 791.1 to 458.8 (LEDD reduction vs. non-reduction, Mann–Whitney U tests, *P*<0.001, Fig. 2B), without apparent complications of movement disorders. Furthermore, randomization of either the reduction or non-reduction groups exerted a significant effect on the primary outcome (Mann–Whitney U tests, *P*<0.001, Fig. 2A). The non-reduction group maintained an ophthalmic level similar to that at baseline (baseline vs. 12 months, VIPD-Q, Mann–Whitney U tests, *P*=0.4759). However, the LEDD reduction group experienced a significant increase in VIPD-Q scores (baseline vs. 12 months, VIPD-Q: Mann–Whitney U tests, *P*<0.001) (PD with reduction vs. PD without reduction, VIPD-Q: Mann–Whitney U tests, *P*<0.001).

Spearman ρ correlations suggested that the LEDD slope was significantly correlated with visual function severity across the entire sample (Fig. 2C, Spearman ρ=0.5323; *P*<0.0001). The greater the LEDD reduction, the higher the VIPD-Q score.

These results were confirmed by multivariate regression analysis (Supplementary Table 4, Supplemental Digital Content 3, http://links.lww.com/JS9/D248).

### RNFL thickness, vessel density, and LEDD reduction

We performed OCT-A (Supplementary Figure 1B, C, Supplemental Digital Content 3, http://links.lww.com/JS9/D248) to determine RNFL thickness and vessel density. Compared with the non-reduction group, the LEDD reduction group reported a decrease in RNFL thickness and vessel density at the endpoint (RNFL: *t*-test, *P*<0.001; VD: *t*-test, *P*<0.001, Fig. 2D and E). In contrast, in the non-reduction group, both parameters were similar to those in patients who did not undergo DBS at the terminal visit (*t*-test, *P*>0.05).

The change in RNFL thickness (slope) was significantly correlated with the slope of the LEDD (Spearman’s ρ=0.5238; *P*<0.001, Fig. 2F). Furthermore, the change in vessel density (slope) was significantly correlated with the slope of the LEDD (Spearman’s ρ=0.6704; *P*<0.001, Fig. 2G). A less pronounced reduction in medication use after STN-DBS was associated with a less pronounced reduction in the RNFL thickness and vessel density.

### STN-DBS and visual saccades

**Figure 3:**
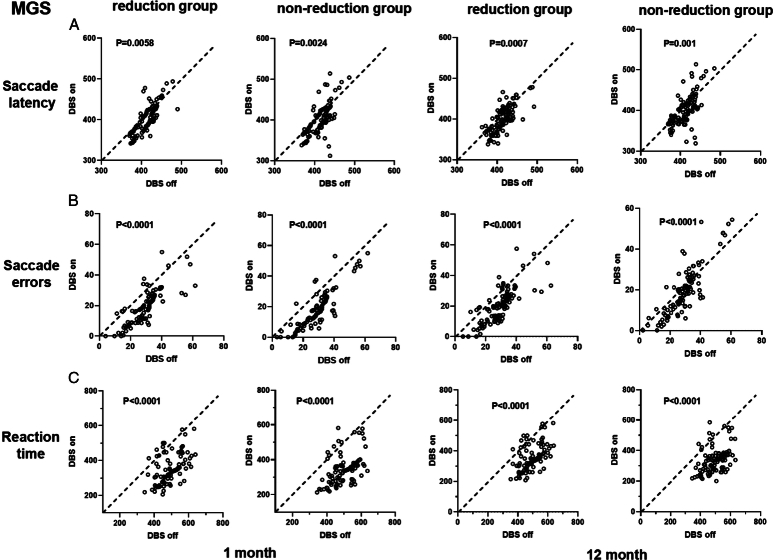
The effect of DBS on the saccade latency, saccade errors, and reaction time of MGS in the reduction group and the non-reduction group. The figures show the effect of DBS on the (A) saccade latency, (B) saccade errors, and (C) reaction time of MGS in the reduction group and the non-reduction group. The horizontal axis displays the DBS-off condition, and the vertical axis displays the DBS-on condition. If the saccade parameter is consistent under STN-DBS-on and DBS-off conditions, the corresponding points will fall on the dotted line passing through the origin. Points under this line demonstrate that the parameter under the DBS-off condition is larger than that under the DBS-on condition, and vice versa. DBS, Deep Brain Stimulation; MGS, Memory-guided Saccade.

Figure 3 shows the effects of DBS on saccade latency (Fig. 3A), saccade errors (Fig. 3B), and reaction time (Fig. 3C). A paired *t*-test between the DBS-on and-off conditions demonstrated that DBS significantly reduced latencies in the MGS (t-test, *P*=0.0058, Fig. 3A). Without DBS, the saccades were more hypometric than those with DBS. DBS significantly improved the accuracy of these saccades (*t*-test, *P*<0.001; Fig. 3B). Without DBS, the reaction time (RT) was longer in patients (*t*-test, *P*<0.001, Fig. 3C). DBS significantly shortened RT in almost all patients in the memory-guide task. Similar results were observed for the VGS (Supplementary Figure 2, Supplemental Digital Content 3, http://links.lww.com/JS9/D248).

No obvious differences in saccadic parameters were observed between the LEDD reduction and non-reduction groups 12 months after implantation. In both groups, STN-DBS reduced the latency, errors, and RT, regardless of LEDD reduction at the final visit (Supplementary Figure 3A, Supplemental Digital Content 3, http://links.lww.com/JS9/D248, *t*-test, *P*>0.05). Furthermore, we observed no significant difference in the saccade latency, errors, or RT of the VGS and MGS tasks between time points 1 and 12 months in the LEDD reduction group (Supplementary Figure 3B, Supplemental Digital Content 3, http://links.lww.com/JS9/D248, t-test, *P*>0.05). Moreover, we reconstructed the location of neurostimulation and calculated the VTA in both LEDD reduction and non-reduction groups by using the software LEAD-DBS Group Analysis. There were no obvious differences in location (Supplementary Figure 4, Supplemental Digital Content 3, http://links.lww.com/JS9/D248) or VTA between the reduction and non-reduction groups (t-test, *P*>0.05, Supplementary Table 5, Supplemental Digital Content 3, http://links.lww.com/JS9/D248).

### Visual function measures and LEDD reduction

The mean visual field deviation and pattern standard deviation were significantly different between groups when the results for both eyes were averaged. The LEDD reduction group demonstrated a significantly more negative slope for mean deviation [mean (SD), −2.14 (1.21) dB/y vs. −0.106 (0.23) dB/y; I-test, *P*<0.001 after FDR correction] and a significantly more positive slope for pattern standard deviation [mean (SD), 1.09 (0.46) dB/y vs. −0.10 (0.56) dB/y; *t*-test, *P*<0.001 after FDR correction] than the control group. These findings indicated gradually worsening visual field thresholds in the LEDD reduction group (Table 2).

Contrast sensitivity testing did not demonstrate significant changes in the LEDD reduction group at the lowest spatial frequency of 1.5 cycles/degree (*t*-test, *P*=0.3194 after FDR correction), 3 cycles/degree (*t*-test, *P*=0.2917 after FDR correction), and the highest spatial frequency of 18 cycles/degree (*t*-test, *P*=0.3194 after FDR correction). The eyes differed significantly at 6 cycles/degree (*t*-test, *P*=0.0288 after FDR correction), and 12 cycles/degree (*t*-test, *P*=0.0288 after FDR correction). Similar results were obtained from the per-protocol analysis (Table 2).

### Long-term follow-up

**Figure 4:**
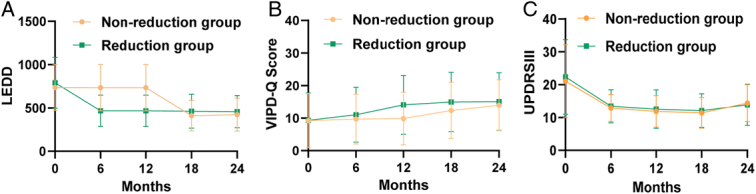
The figures show the changes after the restore of LEDD reduction in the non-reduction group at long-term follow up. (A) The graph shows the mean LEDD of patients with Parkinson’s disease (PD) at 0, 6, 12, 18, and 24 months in reduction and non-reduction groups. (B) The graph shows the mean VIPD-Q score of patients with PD at 0, 12, 18, and 24 months in reduction and non-reduction groups. (C) The graph shows the mean UPDRS-III score of patients with PD at 0, 6, 12, 18, and 24 months in reduction and non-reduction groups. LEDD, levodopa-equivalent dose; UPDRS III, Unified Parkinson’s Disease Rating Scale Section III; VIPD-Q, Visual Impairment in Parkinson’s Disease Questionnaire.

Twelve months postoperatively, most patients with PD in the non-reduction group (98/104) chose to gradually reduce their medication dose, while some patients with PD in the reduction group (18/104) chose to increase their medication dose. The LEDD between the reduction and non-reduction groups demonstrated no noticeable differences at 18 months postoperatively (Mann–Whitney U tests, *P*>0.05; Fig. 4A). However, the prolonged administration of LEDD has extended its protective effects. The VIPD-Q score in the non-reduction group was lower than that in the reduction group 18 months postoperatively (Mann–Whitney U tests, *P*<0.001, Fig. 4B). The protective effects disappeared 24 months postoperatively (Mann–Whitney U tests, *P*>0.05, Fig. 4B). The prolonged use of LEDD did not affect the improvement in UPDRS-III scores (Mann–Whitney U tests, *P*>0.05, Fig. 4C).

## Discussion

STN-DBS decreases the mean dosage of antiparkinsonian drugs by 50%, a step considered essential for diminishing the risk of dyskinesia. No hemorrhage, infection, or other surgical complications were observed during the study. Dyskinesia was present in both groups and the prevalence did not differ significantly between groups.

Whether it is necessary to reduce levodopa for dyskinesia has been a subject of debate. Our experience and the results of a previous study^27^, suggest that levodopa-induced dyskinesia can be alleviated by STN-DBS without reducing the LD. In patients who had dyskinesias before the surgery, we targeted the electrode at the retroposterolateral subthalamic nucleus. In patients who experienced dyskinesias after the surgery, we first changed the stimulation point. A previous study had similar results, showing that STN-DBS improves dyskinesias in PD directly independent of LD reduction^28^. Thus, most participants in our study did not require an LEDD reduction, and only a small minority (21/104) required an LEDD reduction because they could not tolerate the drug-induced dyskinesia.

However, the reduction in medication use is not always beneficial for patients with PD.

This study provides evidence for the negative effects of medication reduction after DBS. DBS implantation did not cause visual dysfunction in patients with PD when the medication was not adjusted by comparing patients who underwent STN-DBS with those who did not undergo STN-DBS. However, worsening of ophthalmologic symptoms was more frequently reported in patients with medication reduction than in those without medication reduction 12 months later.

DBS offers a unique opportunity to understand how the basal ganglia interact with other neural structures^29^. In particular, adaptive DBS could now be used to provide responsive, optimized stimulation for PD^30^. An interesting yet less explored area is the effect of DBS on eye movement and vestibular function. We found that DBS can improve eye movement in a visual saccade task, consistent with previous findings^31^. The potential mechanism may be that STN-DBS could normalize the initiating and inhibiting functions of the basal ganglia (BG), thereby setting the excitability of the superior colliculus (SC) to an appropriate level. Moreover, Beta-band oscillations in the BG are abnormally enhanced in PD^32^, and STN-DBS could decrease these pathologic oscillations. A reduction in the pathologic oscillations by STN-DBS would help maintain the appropriate SC excitability required for saccade initiation and inhibition. Thus, STN-DBS improved the reaction time and accuracy of saccades.

In some studies, LD has been reported as a possible treatment for visual loss in non-arteritic anterior ischemic optic neuropathy or residual amblyopia, and as a protective factor for retinal nerves^16,33^. Increasing the dosage of LD was reported to significantly improve the curative effect in amblyopia^34^. This could be due to LD being essential for normal retinal development and for rescuing retinal morphology and visual function^35^. Therefore, we hypothesized that the deterioration of visual acuity is attributed to another important change: LEDD was reduced significantly after STN-DBS.

This study suggests that LEDD reduction within 12 months of STN-DBS impairs contrast sensitivity and visual field and causes severe progression of visual loss. Participants with reduced LEDD levels presented with a deterioration in visual acuity (contrast and visual field sensitivity); most reported worsening RNFL and vessel density in the ocular fundus. Thinning of the intraretinal layers has been previously described in PD, particularly in the RNFL^33,36,37^. These studies demonstrated that dopamine deficiency in the retina is partly related to contrast sensitivity impairment in PD and could be restored by dopaminergic medication. Future investigations are warranted to better understand why PD causes thinner intraretinal layers.

Physicians recommend simplification of the antiparkinsonian medication regimen, which is constantly observed in the immediate postoperative period after STN-DBS. Nonetheless, no formal studies have been conducted to provide evidence-based criteria to guide physicians in this regard. In our study, LEDD reduction appeared to have a marginal effect on motor symptoms after DBS; however, its influence on non-motor symptoms cannot be ignored. Our study warrants further investigation into the side effects of LEDD reduction after STN-DBS and the optimal levodopa dose to ensure quality of life for Parkinson’s patients while minimizing the risk of visual impairment.

### Strengths and limitations

A major strength of this study is the inclusion of well-characterized individuals who underwent STN-DBS with OCT-A, a Visual Field Analyzer, and eye-tracking system data. Our data provide a novel insight that the reduction in medications after DBS is not always beneficial, which is contrary to the traditional opinion. Our study presents several limitations. Firstly, the long-term effects of medication reduction on visual function were not evaluated. Secondly, the impact of medication reduction on other non-motor symptoms of PD and the dyskinesia ratio was not assessed. Thirdly, the LEDD was utilized to represent a reduction in medication usage, given the challenge of maintaining dopamine agonist monotherapy post-STN-DBS^38^. Finally, connectomic analyses were deficient due to the lacking of functional magnetic resonance imaging (fMRI) data acquired at rest (rs-fMRI) or diffusion-weighted magnetic resonance imaging (dMRI), commonly referred to as “DTI”. Future research should concentrate on elucidating the distinctions between dopamine agonist and LD reductions in terms of visual function. Age of patients, disease duration, implantation position was also considered to influence visual function in previous studies^39,40^. To clarify the effect of confounding factors on our conclusion, we conducted multivariate regression analysis including age, disease duration, gender etc. And results indicated that ratio of visual impairment was obviously related to randomization (Supplementary Table 3, Supplemental Digital Content 3, http://links.lww.com/JS9/D248). In the future, we also intend to use Mendelian randomization to study the causal relationship between LEDD reduction and visual function decline^41^.

## Conclusions

The findings of this prospective cohort study suggest that progressive visual impairment and functional loss correlate with LEDD reduction. We observed greater thinning of the RNFL and lower vessel density in the retina in the LEDD reduction group than in the non-reduction group. Furthermore, we observed a greater decline in functional measures of vision (visual field and contrast sensitivity) in the LEDD reduction group. These findings are consistent with the theoretical models of the chronic effects of LEDD depletion on neurodegeneration. Taken together, the LEDD reduction contributes to visual dysfunction after STN-DBS. Prolonged LEDD administration may be useful in patients with visual dysfunction after STN-DBS.

## Ethical approval

Ethical approval for this study (KYLL-202008-065) was provided by the Ethical Committee of Qilu Hospital of Shandong University, Jinan, China.

## Consent

Written informed consent was obtained from the patient for publication and any accompanying images. A copy of the written consent is available for review by the Editor-in-Chief of this journal on request.

## Source of funding

This work was supported by the National Natural Science Foundation of China [grant number: 81702469], the Natural Science Foundation of Shandong Province [grant number: ZR2023MH321], the Key R&D Program of Shandong Province, China [grant number: 2022ZLGX03], and 2022 Industrial Technology Basic Public Service Platform Project under Grant 2022-189-181.

## Author contribution

C.Z. and W.L. performed the conceptualization; C.Z. performed the resource and funding acquisition; Z.L., J.S., S.M., Z.Z., B.H., and X.L. performed the data analysis and investigation; J.J., X.L., X.M., and J.S. performed the formal analysis and data curation; Q.Q.W. and Q.W. performed the validation; J.J., X.M., and G.Z. performed the methodology; H.W. performed the supervision; Y.H. performed the visualization, software, project administration, and writing—original draft. All authors performed the writing—review and editing and approve of the manuscript.

## Conflicts of interest disclosure

The authors declare no actual or potential conflict of interest.

## Research registration unique identifying number (UIN)

Trial registration number: NCT05901350.

## Guarantor

Chao Zhang and Weiguo Li.

## Data availability statement

The data used for this study, although not available in a public repository, will be made available to other researchers upon reasonable request.

## Provenance and peer review

Not commissioned, externally peer-reviewed.

## Supplementary Material

## References

[1] Valery LF Emma N Tahiya A. Global, regional, and national burden of neurological disorders, 1990-2016: a systematic analysis for the Global Burden of Disease Study 2016. Lancet Neurol 2019;18:459–480. 30879893 10.1016/S1474-4422(18)30499-X PMC6459001

[2] Mok KY. The East Asian Parkinson Disease Genomics Consortium. Lancet Neurol 2021;20:982. 34800411 10.1016/S1474-4422(21)00373-2

[3] Poewe W Seppi K Tanner CM. Parkinson disease. Nat Rev Dis Prim 2017;3:17013. 28332488 10.1038/nrdp.2017.13

[4] Schapira AHV Chaudhuri KR Jenner P. Non-motor features of Parkinson disease. Nat Rev Neurosci 2017;18:435–450. 28592904 10.1038/nrn.2017.62

[5] Hamedani AG Abraham DS Maguire MG. Visual impairment is more common in Parkinson’s disease and is a risk factor for poor health outcomes. Mov Disord 2020;35:1542–1549. 32662528 10.1002/mds.28182 PMC8183672

[6] Elkouzi A Vedam-Mai V Eisinger RS. Emerging therapies in Parkinson disease - repurposed drugs and new approaches. Nat Rev Neurol 2019;15:204–223. 30867588 10.1038/s41582-019-0155-7 PMC7758837

[7] Meng F Hu W Wang S. Utilization, surgical populations, centers, coverages, regional balance, and their influential factors of deep brain stimulation for Parkinson’s disease: a large-scale multicenter cross-sectional study from 1997 to 2021. Int J Surg 2023;109:3322–3336. 37463002 10.1097/JS9.0000000000000603 PMC10651266

[8] Fasano A Daniele A Albanese A. Treatment of motor and non-motor features of Parkinson’s disease with deep brain stimulation. Lancet Neurol 2012;11:429–442. 22516078 10.1016/S1474-4422(12)70049-2

[9] FitzGerald JJ Antoniades CA. Eye movements and deep brain stimulation. Curr Opin Neurol 2016;29:69–73. 26641812 10.1097/WCO.0000000000000276

[10] Khan AN Bronstein A Bain P. Pedunculopontine and subthalamic nucleus stimulation effect on saccades in Parkinson disease. World Neurosurg 2019;126:e219–e231. 30797925 10.1016/j.wneu.2019.02.014

[11] Beylergil SB Murray J Noecker AM. Effects of subthalamic deep brain stimulation on fixational eye movements in Parkinson’s disease. J Comput Neurosci 2021;49:345–356. 33464428 10.1007/s10827-020-00773-2 PMC8286981

[12] Yugeta A Terao Y Fukuda H. Effects of STN stimulation on the initiation and inhibition of saccade in Parkinson disease. Neurology 2010;74:743–748. 20194913 10.1212/WNL.0b013e3181d31e0b

[13] Shaikh AG Antoniades C Fitzgerald J. Effects of deep brain stimulation on eye movements and vestibular function. Front Neurol 2018;9:444. 29946295 10.3389/fneur.2018.00444 PMC6005881

[14] Zhang C Sun J Li Z. Acute visual impairment in a patient with Parkinson’s disease after successful bilateral subthalamic nucleus deep brain stimulation with low-dose levodopa: a case report. Brain Sci 2023;13:103. 36672084 10.3390/brainsci13010103 PMC9857186

[15] Kleiner-Fisman G Herzog J Fisman DN. Subthalamic nucleus deep brain stimulation: summary and meta-analysis of outcomes. Mov Disord 2006;21(suppl 14):S290–S304. 16892449 10.1002/mds.20962

[16] Lyttle DP Johnson LN Margolin EA. Levodopa as a possible treatment of visual loss in nonarteritic anterior ischemic optic neuropathy. Graefe’s Arch Clin Exp Ophthalmol 2016;254:757–764. 26483145 10.1007/s00417-015-3191-z

[17] Hyman MJ Skondra D Aggarwal N. Levodopa is associated with reduced development of neovascular age-related macular degeneration. Ophthalmo Retina 2023;7:745–752. 10.1016/j.oret.2023.04.014 PMC10524303 37146684

[18] Moher D Hopewell S Schulz KF. CONSORT 2010 explanation and elaboration: updated guidelines for reporting parallel group randomised trials. Int J Surg (London, England) 2012;10:28–55. 10.1016/j.ijsu.2011.10.001 22036893

[19] Picillo M Lozano AM Kou N. Programming deep brain stimulation for Parkinson’s disease: The Toronto Western Hospital Algorithms. Brain Stimulation 2016;9:425–437. 26968806 10.1016/j.brs.2016.02.004

[20] Jost ST Kaldenbach M-A Antonini A. Levodopa dose equivalency in Parkinson’s disease: updated systematic review and proposals. Mov Disord 2023;38:1236–1252. 37147135 10.1002/mds.29410

[21] Borm C Visser F Werkmann M. Seeing ophthalmologic problems in Parkinson disease: results of a visual impairment questionnaire. Neurology 2020;94:e1539–e1547. 32161030 10.1212/WNL.0000000000009214 PMC7251522

[22] Gilmore CS Lim KO Garvin MK. Association of optical coherence tomography with longitudinal neurodegeneration in veterans with chronic mild traumatic brain injury. JAMA Netw Open 2020;3:e2030824. 33351088 10.1001/jamanetworkopen.2020.30824 PMC7756235

[23] Horn A Reich M Vorwerk J. Connectivity Predicts deep brain stimulation outcome in Parkinson disease. Ann Neurol 2017;82:67–78. 28586141 10.1002/ana.24974 PMC5880678

[24] Garza R Amil AS Nowacki A. Patient-Specific anisotropic volume of tissue activated with the lead-DBS toolbox. Annu Int Conf IEEE Eng Med Biol Soc 2021;2021:6285–6288. 34892550 10.1109/EMBC46164.2021.9629810

[25] Ewert S Plettig P Li N. Toward defining deep brain stimulation targets in MNI space: A subcortical atlas based on multimodal MRI, histology and structural connectivity. Neuroimage 2018;170:271–282. 28536045 10.1016/j.neuroimage.2017.05.015

[26] Meoni S Bradi AC Wadia P. Dyspnea after subthalamic deep brain stimulation in Parkinson’s disease: a case-control study. J Neurol 2020;267:3054–3060. 32524258 10.1007/s00415-020-09976-0

[27] Kim JH Chang WS Jung HH. Effect of subthalamic deep brain stimulation on levodopa-induced dyskinesia in Parkinson’s disease. Yonsei Med J 2015;56:1316–1321. 26256974 10.3349/ymj.2015.56.5.1316 PMC4541661

[28] Mossner JM Patil PG Chou KL. Subthalamic nucleus deep brain stimulation improves dyskinesias in Parkinson’s disease beyond levodopa reduction. J Neural Transm (Vienna) 2019;126:1479–1483. 31494731 10.1007/s00702-019-02076-y

[29] Krauss JK Lipsman N Aziz T. Technology of deep brain stimulation: current status and future directions. Nat Rev Neurol 2021;17:75–87. 33244188 10.1038/s41582-020-00426-z PMC7116699

[30] Wang S Zhu G Shi L. Closed-loop adaptive deep brain stimulation in Parkinson’s disease: procedures to achieve it and future perspectives. J Parkinsons Dis 2023;13:453–471. 37182899 10.3233/JPD-225053 PMC10357172

[31] Yugeta A Hutchison WD Hamani C. Modulation of Beta oscillations in the subthalamic nucleus with prosaccades and antisaccades in Parkinson’s disease. J Neurosci 2013;33:6895–6904. 23595748 10.1523/JNEUROSCI.2564-12.2013 PMC6618858

[32] van Wijk BCM Neumann W-J Schneider G-H. Low-beta cortico-pallidal coherence decreases during movement and correlates with overall reaction time. Neuroimage 2017;159:1–8. 28712991 10.1016/j.neuroimage.2017.07.024 PMC5678295

[33] Satue M Seral M Otin S. Retinal thinning and correlation with functional disability in patients with Parkinson’s disease. Br J Ophthalmol 2014;98:350–355. 24276697 10.1136/bjophthalmol-2013-304152

[34] Wang S-P Li Q-X Li S. Systematic evaluation of levodopa effect on visual improvement in amblyopia: a meta-analysis. Clin Neuropharmacol 2020;43:20–25. 31738189 10.1097/WNF.0000000000000372

[35] Lee H Scott J Griffiths H. Oral levodopa rescues retinal morphology and visual function in a murine model of human albinism. Pigment Cell Melanoma Res 2019;32:657–671. 30851223 10.1111/pcmr.12782 PMC6766973

[36] Hajee ME March WF Lazzaro DR. Inner retinal layer thinning in Parkinson disease. Arch Ophthalmol (Chicago, Ill: 1960) 2009;127:737–741. 10.1001/archophthalmol.2009.106 19506190

[37] Garcia-Martin E Larrosa JM Polo V. Distribution of retinal layer atrophy in patients with Parkinson disease and association with disease severity and duration. Am J Ophthalmol 2014;157:470–478.e472. 24315296 10.1016/j.ajo.2013.09.028

[38] Picillo M Phokaewvarangkul O Poon Y-Y. Levodopa versus dopamine agonist after subthalamic stimulation in Parkinson’s disease. Mov Disord 2021;36:672–680. 33165964 10.1002/mds.28382 PMC8048876

[39] Petry-Schmelzer JN Krause M Dembek TA. Non-motor outcomes depend on location of neurostimulation in Parkinson’s disease. Brain 2019;142:3592–3604. 31553039 10.1093/brain/awz285

[40] Cvekl A Vijg J. Aging of the eye: lessons from cataracts and age-related macular degeneration. Ageing Res Rev 2024;99:102407. 38977082 10.1016/j.arr.2024.102407 PMC11288402

[41] Sekula P Del Greco M F Pattaro C. Mendelian randomization as an approach to assess causality using observational data. J Am Soc Nephrol 2016;27:3253–3265. 27486138 10.1681/ASN.2016010098 PMC5084898

