# Supplementary figures and images for: The reduction of LEDD leads to visual dysfunction in patients with PD after STN-DBS: a randomized clinical trial
Source: Int J Surg. 2024 Aug 5;111(1):650–60. doi: 10.1097/JS9.0000000000002018 (PMC11745593; doi:10.1097/JS9.0000000000002018)

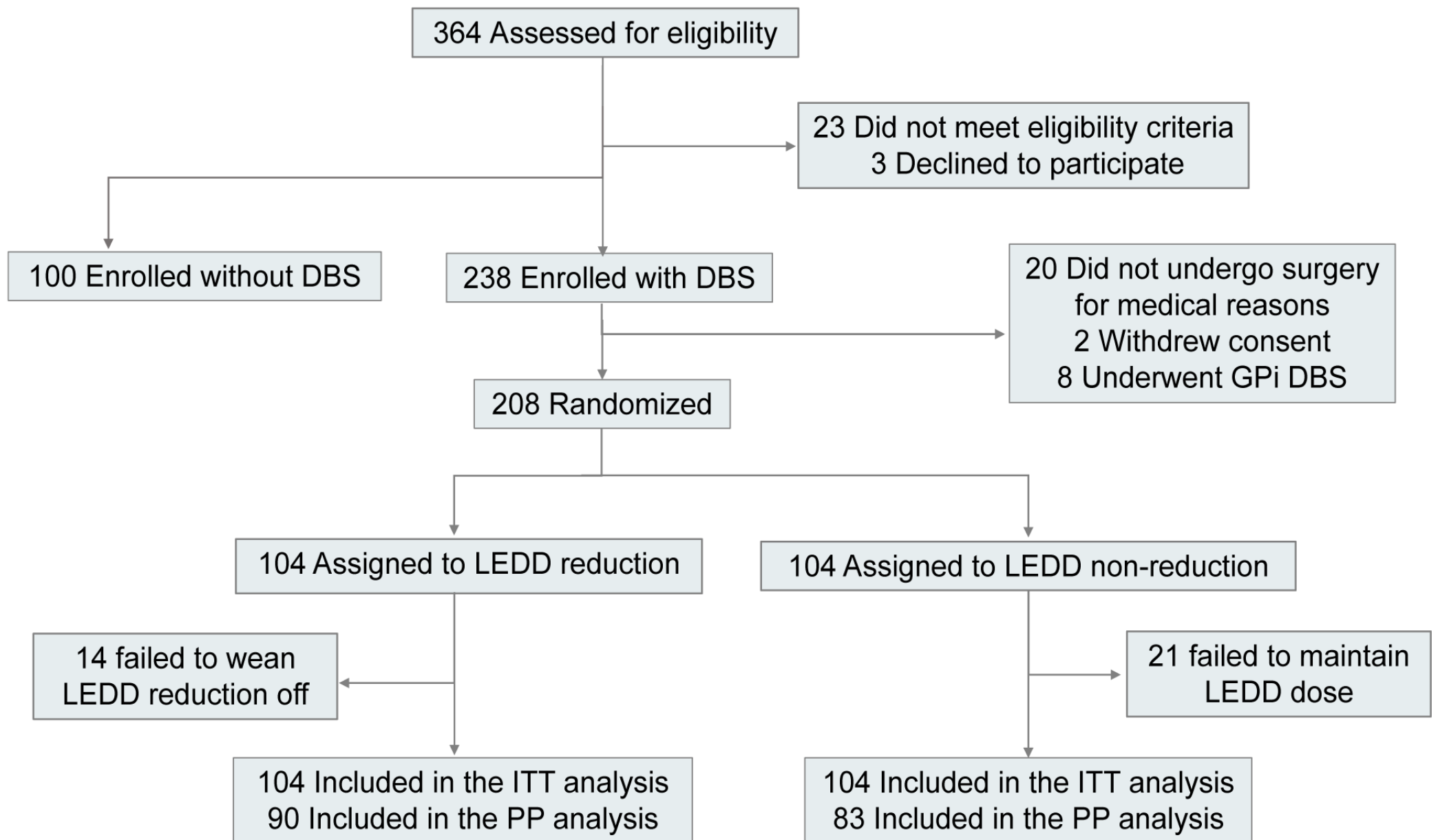

Supplement: Supplementary file 2 [file js9-111-0650-s002.pdf]
