# Supplementary material for: The reduction of LEDD leads to visual dysfunction in patients with PD after STN-DBS: a randomized clinical trial
Source: Int J Surg. 2024 Aug 5;111(1):650–60. doi: 10.1097/JS9.0000000000002018 (PMC11745593; doi:10.1097/JS9.0000000000002018)
Supplement: Supplementary file 3 [file js9-111-0650-s003.pdf]

Supplementary Contents

Supplementary Figure 1. Visual Evaluation Tools

Supplementary Figure 2. The effect of DBS on the saccade latency, saccade errors, and reaction time of VGS in the LEDD reduction group and the non-reduction group
Supplementary Figure 3. The effect of LEDD reduction on the saccadic parameter
Supplementary Figure 4 . Lead location in the LEDD reduction and the non-reduction group

Supplementary Table 1. Number of patients included in each center
Supplementary Table 2. Baseline characteristics of the PD with STN-DBS and PD without STN-DBS cohort.
Supplementary Table 3. Three-month post-operative stimulation parameters for the intention-to-treat and per-protocol population according to randomization.
Supplementary Table 4. Predictors of baseline and 12 months visual impairment: multivariate regression (all subjects).
Supplementary Table 5. Distribution of VTA in the LEDD reduction and the non-reduction group

Supplementary Figure 1. Visual Evaluation Tools

A

Visual Impairment in Parkinson's Disease Questionnaire (VIPD-Q)

Ocular surface

1. I have blurry vision when I read or work on a computer.
2. I have a burning sensation or gritty feeling in my eyes
3. I have mucus/slime or particles in my eyes or eyelids.
4. I have watery eyes.

Intraocular

1. When I read, some letters disappear
2. Lines that should be straight appear to be wavy or blurred.
3. I won't go out alone in the evening or at night because my night vision is insufficient.
4. When I drive at night, the oncoming headlights cause more glare than before.

Oculomotor

1. Quick movements are hard to follow with my eyes.
2. I have double vision.
3. I can read better with one eye closed.
4. I have trouble with depth perception. I find it hard to say which one of 2 objects is closer.

Optic nerve

1. Colors seem to be paler than before.
2. I can't read plain text on a colored or gray background.
3. I run into objects or people or feel that parts of my visual field are missing.
4. I have problems with rapid changes of light intensity.
5. I see things that other people do not see (hallucinations).

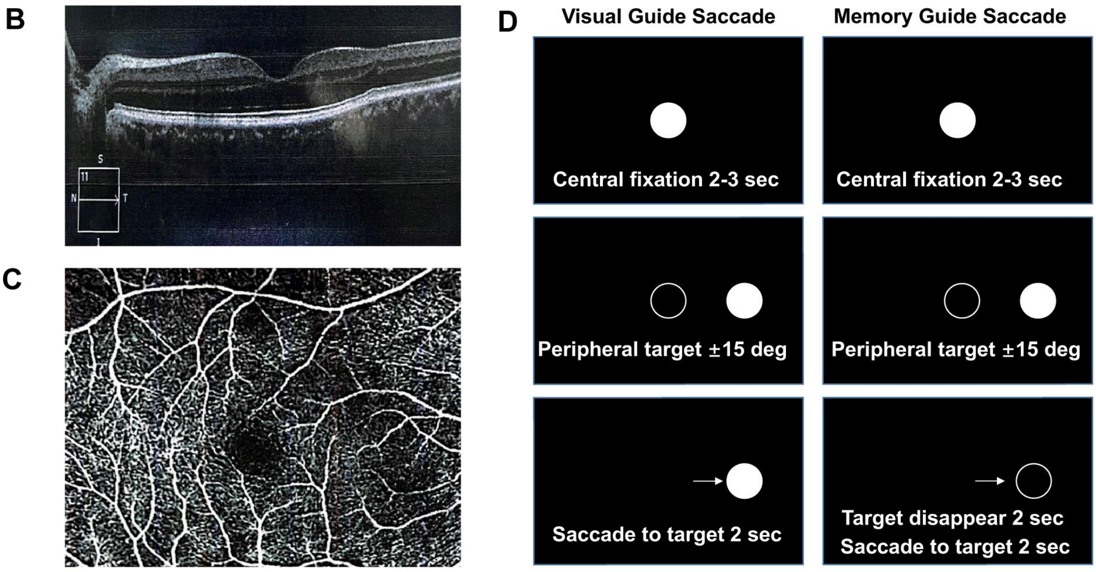

Supplementary Figure 1. Visual Evaluation Tools (A) The VIPD-Q screening questionnaire was used to assess ophthalmologic symptoms. (B) The RNFL and retinal thickness were measured. (C) Ocular fundus photography was performed. (D) VGS and MGS

Supplementary Figure 2. The effect of DBS on the saccade latency, saccade errors, and reaction time of VGS in the LEDD reduction group and the non-reduction group

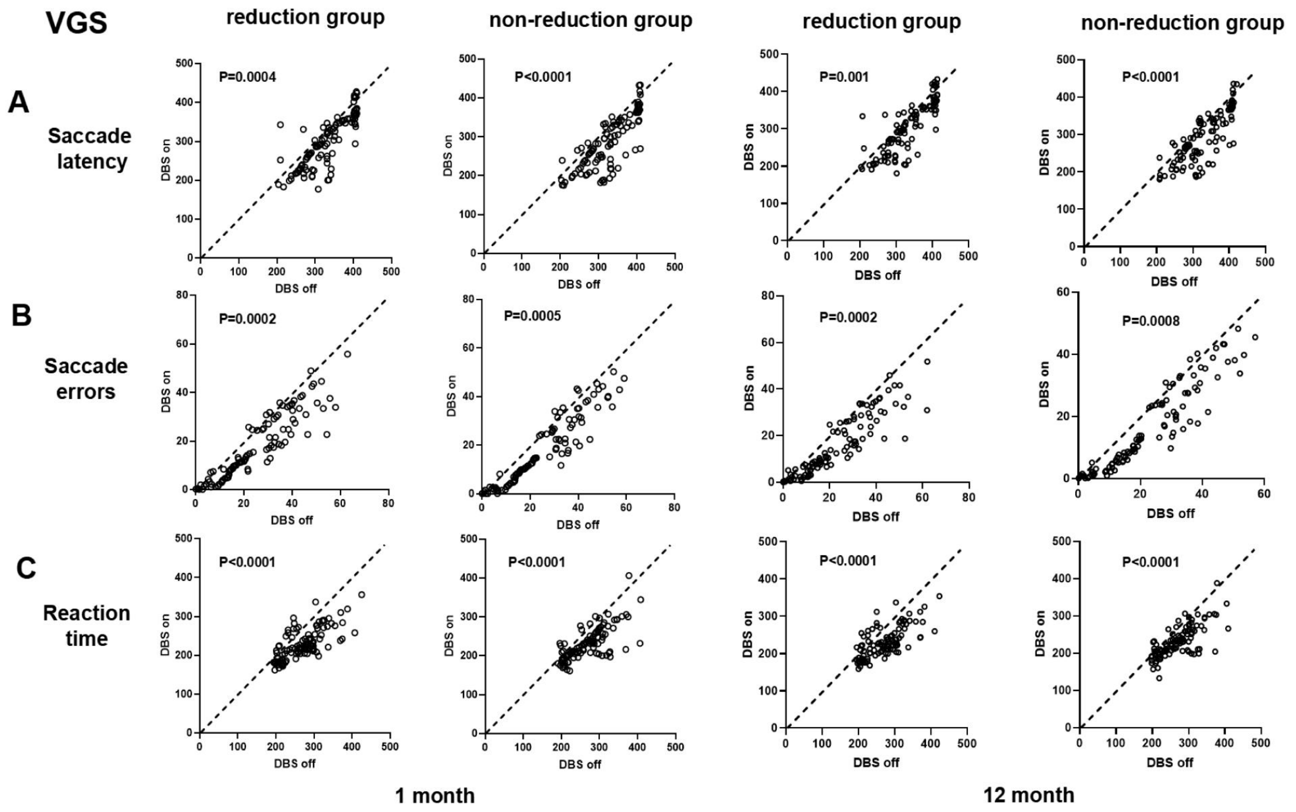

Supplementary Figure 2. The figures show the effect of DBS on the (A) saccade latency, (B) saccade errors, and (C) reaction time of VGS in the LEDD reduction group and the non-reduction group. The horizontal axis displays the DBS-off condition, and the vertical axis displays the DBS-on condition. If the saccade parameter is consistent under STN DBS-on and DBS-off conditions, the corresponding points will fall on the dotted line passing through the origin. Points under this line demonstrate that the parameter under the DBS-off condition is larger than that under the DBS-on condition, and vice versa.

Supplementary Figure 3. The effect of LEDD reduction on the saccadic parameter

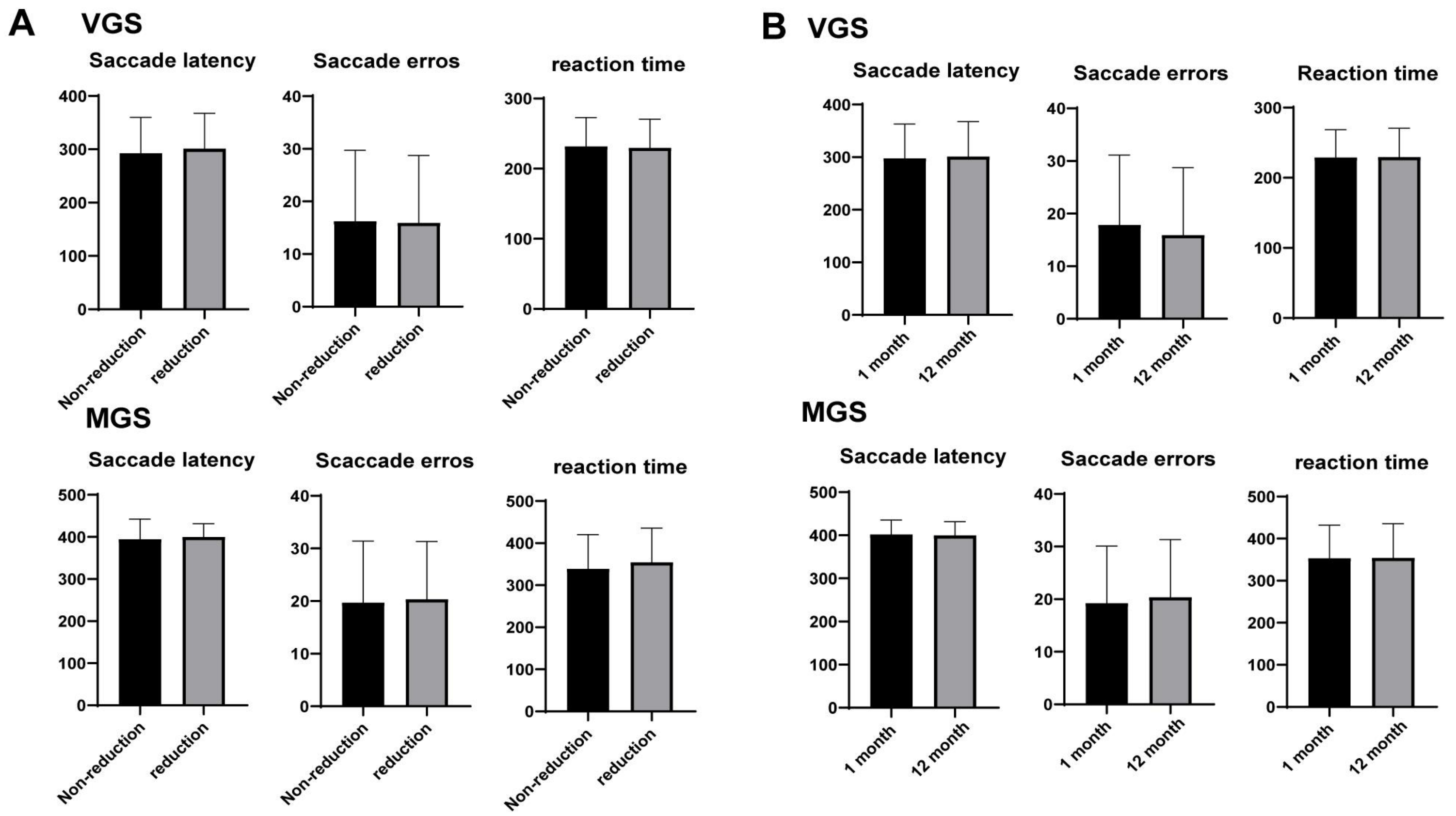

Supplementary Figure 3. The figures show the effect of LEDD reduction on the saccadic parameters. (A) STN-DBS was capable of reducing latency, errors, and reaction time regardless of whether LEDD was reduced or not at the final visit. (B) There was also no significant difference in saccade latency, errors, or reaction time of the VGS and MGS tasks between time points 1 and 12 months in the LEDD reduction group.

Supplementary Figure 4. Lead location in the LEDD reduction and the non-reduction group

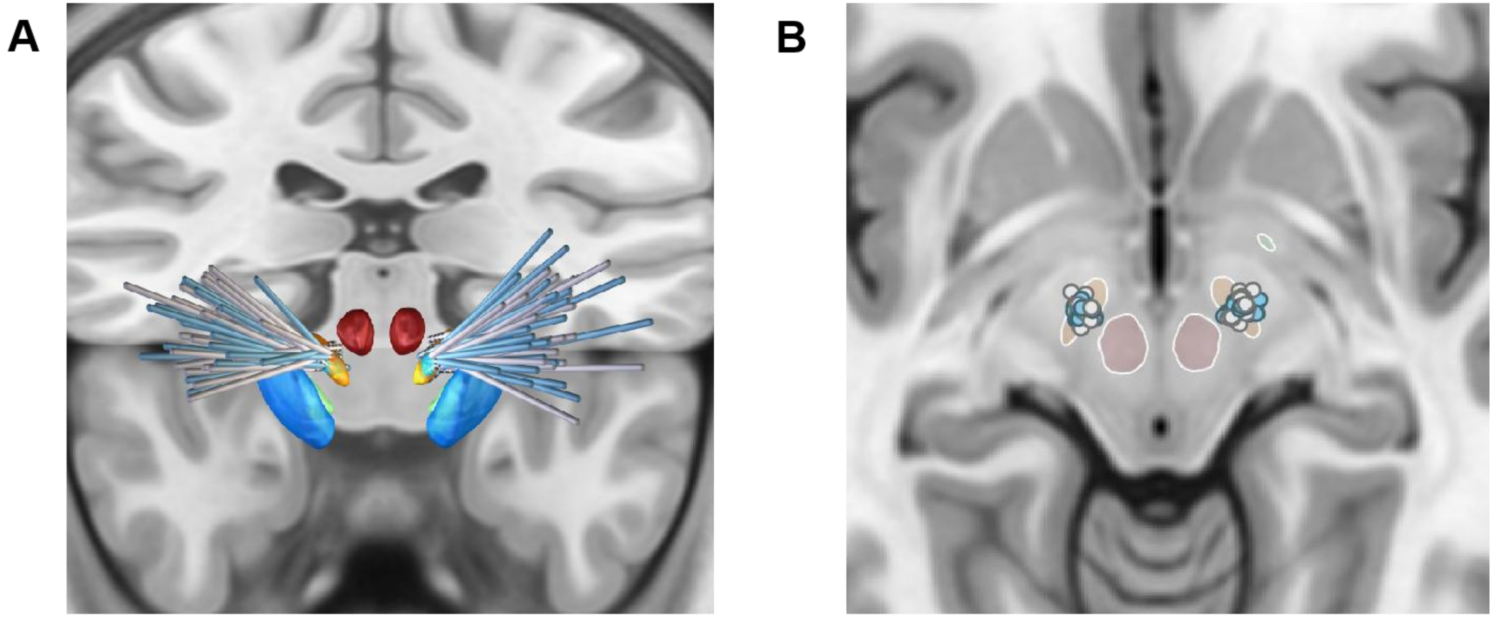

Supplementary Figure 4. The figures show the location of electrode in the LEDD reduction (marked by white) and the non-reduction group (marked by blue). (A) Reconstructed electrodes show the typical frontal view of bilateral lead positions in standard stereotactic space projected on the STN as implemented in the DISTAL atlas. (B) The location of electrodes' distribution at the level of locus ruber.

Supplementary Table 1. Number of patients included in each center

	Center 1	Center 2	Center 3
PD with STN-DBS (n=208) (n)	128	52	28
PD without STN-DBS Control (n=100) (n)	64	24	12
Intention-to-treat			
PD with STN-DBS (LEDD reduction) (n=104) (n)	66	26	12
PD with STN-DBS (LEDD non-reduction) (n=104) (n)	66	26	12
Per-protocol			
PD with STN-DBS (LEDD reduction) (n=90) (n)	58	22	10
PD with STN-DBS (LEDD non-reduction) (n=83) (n)	54	20	9

Supplementary Table 2. Baseline characteristics of the PD with STN-DBS and PD without STN-DBS cohort.

	PD with STN-DBS (n=208)	PD without STN-DBS Control (n=100)	*P* value
Men, n (%)	110, (52.8)	56, (56.0)	0.6075
Age, (yr)	62.28 ± 9.10	63.84 ± 10.31	0.3107
Disease duration, (yr)	9.16 ± 4.67	9.32 ± 4.80	0.2287
NMSS	55.82 ± 42.75	52.51 ± 45.38	0.2106
UPDRS-III (med on)	21.69 ± 11.25	21.75 ± 10.82	0.8321
MoCA	25.57 ± 1.41	25.84 ± 1.96	0.8879
MMSE	27.91 ± 1.61	27.85 ± 1.66	0.6095
HAD-A	5.63 ± 3.17	5.17 ± 2.36	0.2039
HAD-D	4.82 ± 2.55	5.14 ± 4.35	0.3992
Uses visual aid, n (%)	62, (29.8)	32, (32)	0.6956
LEDD (mg)	763.2 ± 281.3	818.5 ± 444.8	0.7858

Abbreviations: NMSS, Non-Motor Symptoms Scale; UPDRS III, Unified Parkinson's Disease Rating Scale Section III; MoCA, Montreal Cognitive Assessment; MMSE, mini-mental state examination; HAD-A, Hospital Anxiety Depression Scale—anxiety subscore; HAD-D, Hospital Anxiety Depression Scale—depression subscore; LEDD, levodopa equivalent daily dose

Supplementary Table 3. Three-month post-operative stimulation parameters for the intention-to-treat and per-protocol population according to randomization

	Intention-to-treat			Per-protocol		
	LEDD reduction (N = 104)	LEDD non-reudction (N = 104)	*P* value	LEDD reduction (N = 90)	LEDD non-reudction (N = 83)	*P* value
Right amplitude, V	2.96 ± 0.82	2.89 ± 1.2	0.624	2.84 ± 0.82	2.71 ± 1.19	0.401
Right pulse width, μsec	61.5 ± 7.55	62 ± 3.8	0.547	61.8 ± 8.13	61.5 ± 6.0	0.784
Right frequency, Hz	130.54 ± 22.32	132.41 ± 30.26	0.613	132.54 ± 27.16	135.44 ± 31.51	0.517
Left amplitude, V	3.07 ± 0.78	2.88 ± 1.46	0.243	3.06 ± 0.85	2.78 ± 1.44	0.118
Left pulse width, μsec	63 ± 11.56	62 ± 4.0	0.405	62 ± 15.47	63.2 ± 8.0	0.528
Left frequency, Hz	131.48 ± 23.43	136.38 ± 27.14	0.165	132.56 ± 26.38	133.42 ± 20.15	0.811

Data are in mean ± standard deviation, unless otherwise specified. Abbreviations: DA: dopamine receptor agonist; LD: Levodopa.

Supplementary Table 4. Predictors of baseline and 12 months visual impairment: multivariate regression (all subjects).

Independent variable	OR (95%CI)	*P value*
Age	0. 93 (0.86 to 1.22)	0.367
Disease duration	0. 92 (0.84 to 1.32)	0.566
UPDRS-III	1. 22 (0.98 to 1.32)	0.231
Gender	1.02 (0.89 to 1.27)	0.519
LEDD	0. 39 (0.29 to 1.44)	0.023
Randomization	5.1 (1.17 to 11.54)	0.029

Abbreviations: CI: confidence interval; LEDD: levodopa equivalent daily dose; OR: odds ratio.

Supplementary Table 5. Distribution of VTA in the LEDD reduction and the non-reduction group**.**

STN location	LEDD reduction group (mm3)	LEDD non-reduction (mm3)	*P* value
Sensorimotor	56 ± 12	52 ± 18	0.067
Assicuatuve	52 ± 18	55 ± 16	0.216
Limbic	55 ± 14	52 ± 20	0.223

Abbreviations: STN: subthalamic nucleus; LEDD: levodopa equivalent daily dose; VTA: volume of tissue activated.
